# Randomization to Screening for Prostate, Lung, Colorectal and Ovarian Cancers and Thyroid Cancer Incidence in Two Large Cancer Screening Trials

**DOI:** 10.1371/journal.pone.0106880

**Published:** 2014-09-05

**Authors:** Thomas J. O'Grady, Cari M. Kitahara, A. Gregory DiRienzo, Francis P. Boscoe, Margaret A. Gates

**Affiliations:** 1 University at Albany, School of Public Health, Rensselaer, New York, United States of America; 2 Division of Cancer Epidemiology and Genetics, National Cancer Institute, National Institutes of Health, Rockville, Maryland, United States of America; Cedars Sinai Medical Center, United States of America

## Abstract

**Background:**

Thyroid cancer incidence has increased significantly over the past three decades due, in part, to incidental detection. We examined the association between randomization to screening for lung, prostate, colorectal and/or ovarian cancers and thyroid cancer incidence in two large prospective randomized screening trials.

**Methods:**

We assessed the association between randomization to low-dose helical CT scan versus chest x-ray for lung cancer screening and risk of thyroid cancer in the National Lung Screening Trial (NLST). In the Prostate Lung Colorectal and Ovarian Cancer Screening Trial (PLCO), we assessed the association between randomization to regular screening for said cancers versus usual medical care and thyroid cancer risk. Over a median 6 and 11 years of follow-up in NLST and PLCO, respectively, we identified 60 incident and 234 incident thyroid cancer cases. Cox proportional hazards regression was used to calculate the cause specific hazard ratios (HR) and 95% confidence intervals (CI) for thyroid cancer.

**Results:**

In NLST, randomization to lung CT scan was associated with a non-significant increase in thyroid cancer risk (HR  = 1.61; 95% CI: 0.96–2.71). This association was stronger during the first 3 years of follow-up, during which participants were actively screened (HR  = 2.19; 95% CI: 1.07–4.47), but not subsequently (HR  = 1.08; 95% CI: 0.49–2.37). In PLCO, randomization to cancer screening compared with usual care was associated with a significant decrease in thyroid cancer risk for men (HR  = 0.61; 95% CI: 0.49–0.95) but not women (HR  = 0.91; 95% CI: 0.66–1.26). Similar results were observed when restricting to papillary thyroid cancer in both NLST and PLCO.

**Conclusion:**

Our study suggests that certain medical encounters, such as those using low-dose helical CT scan for lung cancer screening, may increase the detection of incidental thyroid cancer.

## Introduction

Thyroid cancer incidence has increased dramatically in recent years while increases in thyroid cancer mortality have been modest [1], [2]. Established risk factors for thyroid cancer include exposure to ionizing radiation, primarily in childhood, as well as past history of thyroid nodules or thyroid disorders [3]. Increased medical scrutiny may lead to the detection of small, non-aggressive, thyroid carcinomas that, if left untreated, might never affect the health of the patient. A large reservoir of subclinical thyroid tumors present in the general population supports the possibility that some of the increase in thyroid cancer incidence may be due to incidental diagnosis of these subclinical tumors [4], [5]. The potential role of diagnostic imaging in the detection of incidental thyroid cancers comes from literature reporting that 16% of all diagnostic CT scans and MRIs show incidental thyroid nodules, largely less than 1.5 cm [6], [7], and that 60% of thyroid cancers are incidentally detected by a doctor via medical imaging or during treatment for a benign thyroid disorder [8]. Registry data from Wisconsin [9] and New Jersey [10] indicate that higher socioeconomic status, higher education, and greater healthcare access and utilization are associated with increased thyroid cancer rates.

The current thyroid cancer trends, and the recent findings regarding these trends, highlight the necessity to study in greater detail the impact that various medical encounters have on the diagnosis of thyroid carcinomas at a population level. The present study is the first of its kind to assess the impact of lung, prostate, colorectal, and ovarian cancer screening on thyroid cancer incidence in two large randomized screening trials of men and women conducted by the National Institutes of Health (NIH), the National Lung Screening Trial (NLST) and Prostate, Lung, Colorectal, and Ovarian (PLCO) Cancer Screening Trial. We hypothesized that the type of medical encounter an individual has may be important to the detection of incidental thyroid cancer. Specifically, we hypothesized that individuals randomized to receive low-dose helical CT scan, as compared with standard chest x-ray, for lung cancer screening are more likely to be diagnosed with thyroid cancer because CT scans are highly sensitive and the imaging field often includes the thyroid gland. Additionally, we predicted that thyroid cancer incidence may be associated with the use of chest x-ray for lung cancer screening but would not be associated with randomization to other forms of cancer screening (as compared to standard medical care) such as flexible sigmoidoscopy for colorectal cancer, digital rectal exam and serum prostate specific antigen for prostate cancer, and transvaginal ultrasound and CA125 for ovarian cancer.

## Methods

### Study Population

Study participants were enrolled in one of two large U.S. based prospective randomized screening trials: NLST [11], [12] and PLCO [13]. The NLST and PLCO study protocol was approved by the Institutional Review Board of the National Cancer Institute and all participating institutions. Additionally use of the data for this study was approved by the Institutional Review Boards of the National Cancer Institute and the University at Albany. All participants of NLST and PLCO provided written informed consent upon enrollment.

Eligible participants for this analysis responded to a baseline questionnaire, were followed for cancer incidence, had no history of cancer other than non-melanoma skin cancer at baseline, and were not missing a diagnosis date for any cancer diagnosed during follow-up. We excluded 204 individuals from NLST who were not eligible for the screening trial because they were not between the ages of 55 and 74 (n = 12), were never smokers (n = 36), had a CT scan within 18 months of enrollment (n = 71), were part of another screening or prevention trial (n = 29), previously had lung cancer (n = 2), previously had a portion of their lung removed (n = 8), had cancer within the past 5 years (n = 24), or had other health related complications (n = 22). From PLCO we excluded individuals without a baseline questionnaire completed (n = 4,919), who had a personal history of cancer (n = 1,960) or their personal history of cancer could not be determined (n = 4,933), and without any follow-up time (n = 692). Our final study population consisted of 53,248 (31,423 men/21,825 women) participants in NLST and 142,394 (71,787 men/70,607 women) in PLCO.

### Exposure Assessment

In each study the primary exposure of interest was randomization to the intervention arm of the trial. The randomization procedures for each study are described below. In NLST interested participants who contacted participating screening centers were assessed for eligibility. Participants who were deemed eligible and had signed informed consent were block randomized in equal proportions to receive low-dose helical CT scans (intervention group) or chest radiography (control group). Participants were offered screening a total of three times, once at the beginning of years one through three.

Each screening center in PLCO established its own procedures for identifying and recruiting participants based on guidelines set forth by the NCI. Individuals meeting those eligibility criteria were block randomized in equal proportions to the screened and control arms. Men in the screening arm received chest x-ray, flexible sigmoidoscopy, PSA, and digital rectal exam while women received chest x-ray, flexible sigmoidoscopy, CA125, and transvaginal ultrasound. Men and women in the control arm were instructed to follow their usual medical care practices. Participants in the intervention arm were offered screening a total of six times, once at the beginning of years one through six.

Covariate information in each trial came from a self-administered baseline questionnaire to assess general demographic information, lifestyle factors, and health characteristics.

### Cancer Ascertainment

Participants accrued time in each study from the date of baseline questionnaire completion to the date of diagnosis of any cancer other than non-melanoma skin cancer, death, or last date of follow-up, whichever came first. Information on incident cancers in each trial was obtained through self-report and death certificates. Medical record abstraction was used to verify individuals with self-reported cancer. Self-reported cancers subsequently de-confirmed during medical record review were excluded from our analysis. The primary outcome of interest was thyroid cancer (International Classification of Disease for Oncology, Third Edition (ICD-O-3), topography code C73.9) [14]. We also evaluated papillary thyroid cancer (ICD-O-3 morphology codes: 8050, 8052, 8260, 8340-8344 [14]) separately because incidental papillary micro-carcinomas are the primary histology documented [15].

### Statistical Analysis

Cox proportional hazards regression [16] with person-time as the underlying time metric was used to estimate the cause specific hazard ratios (HR) and corresponding 95% confidence intervals (CI) for thyroid cancer. We assessed and verified, using cumulative sums of martingale residuals [17], that there was no violation of the proportional hazards assumption. Potential confounders were analyzed using a backward elimination method in which we removed the least significant covariate in the model and assessed whether this changed the main exposure HR by more than 10%. In each study we assessed age (continuous), sex, ethnicity (Hispanic or Non-Hispanic), race (White, Black, other), body mass index (<18.5, 18.5-<25, 25-<30, 30+), education (high school or less, some college, college or post graduate), smoking (NLST: former or current, PLCO: never, former, current), and marital status (married or not married) as potential confounders. Additionally, we compared complete case analysis with using a missing indicator variable to handle missing data. We then performed a sex-stratified analysis that was minimally adjusted by age and tested for interaction, using the likelihood ratio test. Because we had different hypotheses for NLST and PLCO we conducted separate analyses for each study.

After we assessed randomization to the intervention arm over the entire follow-up period, we fit a Cox proportional hazards model with a time-dependent covariate for the exposure variable (randomization arm) to estimate two associations: one with randomization to intervention during active screening (0–3 years in NLST and 0–6 years in PLCO) and the other from the end of active screening to the end of the study follow-up period. Finally, to assess whether the length of time of active screening impacted the association between randomization to intervention and thyroid cancer risk we evaluated the association of intervention in PLCO with a cut point of three years for a more direct comparison to NLST.

SAS software version 9.3 (SAS Institute, Cary, NC) was used to complete all statistical analysis. All reported p-values were based on two-sided tests and an alpha level of 0.05.

## Results

The mean age of participants at baseline was 61.6 years for men and 61.2 years for women in both arms of NLST. The NLST cohort was over 90% non-Hispanic White and there was a higher percentage of men (59%) than women. Of the 60 thyroid cancer cases 36 (60%) were of papillary histology. The mean age of PLCO participants was 62.7 years for men and 62.5 years for women in both arms. There was roughly the same number of men as women (50.7% men/49.3% women), less than 10% of the participants were current smokers, the cohort was over 85% non-Hispanic White and 77% of the 234 incident thyroid cancers were of papillary histology (Tables 1 & 2). The median (range) of follow-up for participants in NLST and PLCO was 6 years (0 to 7 years) and 11 years (0 to 13 years) respectively. Including an indicator variable for missing values of potential confounders had little effect on the HRs calculated using individuals with complete confounder information. Because we found no evidence of confounding by any variable considered we minimally adjusted the overall analysis by age and sex (all individuals had information on age and sex).

**Table 1:** Baseline characteristics of participants in NLST by randomization group.

		Male N = 31,423		Female N = 21,825	
		Control Arm	Intervention Arm	Control Arm	Intervention Arm
	N =	15,698	15,725	10,923	10,902
Number of thyroid cancer cases		11	17	12	20
Number of papillary thyroid cancer cases		7	9	9	11
**Parameter**	**Characteristics**				
Age^1^		61.6 (5.1)	61.6 (5.1)	61.2 (4.9)	61.2 (4.9)
Race	White (%)	90.6	90.6	91.1	91.3
	Black (%)	4.1	4.1	4.8	5.0
Ethnicity^2^	Hispanic (%)	1.8	2.0	1.6	1.5
	Non-Hispanic (%)	97.5	97.4	97.4	97.9
Currently Married (%)		75.9	76.1	53.7	53.6
BMI (kg/m^2^)^1^		28.2 (4.6)	28.2 (4.6)	27.5 (5.7)	27.5 (5.6)
Smoking History	Never Smoker (%)	0	0	0	0
	Former Smoker (%)	52.8	53.4	50.1	49.6
	Current Smoker (%)	47.2	46.6	49.9	50.4
Education (% College or Post-Grad)		35.4	35.4	24.4	24.6
History of Diabetes (% Yes)		11.1	11.4	7.7	7.4

1 Mean and standard deviation.

2 Values do not sum to 1 because of missing values.

**Table 2:** Baseline characteristics of participants in the PLCO by randomization group.

		Male N = 71,787		Female N = 70,607	
		Control Arm	Intervention Arm	Control Arm	Intervention Arm
	N =	35,351	36,463	35,256	35,324
Number of thyroid cancer cases		53	34	77	70
Number of papillary thyroid cancer cases		41	22	65	53
**Parameter**	**Characteristics**				
Age^1^		62.7 (5.3)	62.7 (5.3)	62.5 (5.4)	62.5 (5.4)
Race	White (%)	88.2	88.2	88.4	88.5
	Black (%)	4.6	4.6	5.8	5.8
Ethnicity	Hispanic (%)	2.4	2.4	1.8	1.8
	Non-Hispanic (%)	97.6	97.6	98.2	98.2
Currently Married (%)		82.8	82.9	69.1	69.3
BMI (kg/m^2^)		27.5 (4.1)	27.6 (4.2)	27.1 (5.7)	27.2 (5.6)
Smoking History^3^	Never Smoker (%)	36.3	36.7	55.9	56.1
	Former Smoker (%)	52.0	51.6	34.5	34.3
	Current Smoker (%)	11.6	11.8	9.6	9.6
Education (% College or Post-Grad)		40.4	40.9	29.7	29.8
Family History Cancer (% Yes)		51.7	51.7	59.2	59.2
History of Diabetes (% Yes)		9.0	9.1	6.4	6.4
Ever used Birth Control (% Yes)		n/a	n/a	54.6	54.0
Ever used female hormones (% Yes)		n/a	n/a	66.7	67.1
Ever been pregnant (% Yes)		n/a	n/a	92.4	92.6
Have Benign Breast Disease (%Yes)		n/a	n/a	28.0	27.8

1 Mean and standard deviation.

2 Values do not sum to 1 because of missing values.

3 Does not sum to 1 because of rounding.

We observed a non-significant increase (HR  = 1.61; 95% CI: 0.96–2.71) in thyroid cancer risk for the intervention arm in NLST while in PLCO we observed a non-significant decrease in risk (HR  = 0.79; 95% CI: 0.61–1.02), in models adjusted for age and sex. Evaluating thyroid cancer risk separately for men and women did not change the direction of the association in either cohort and found no evidence of effect modification by sex in NLST (p = 0.89) or PLCO (p = 0.15). The association between randomization to the intervention arm of PLCO and thyroid cancer risk was statistically significant inverse for men (HR  = 0.61; 95% CI: 0.40–0.95). Associations observed for papillary thyroid cancer only were similar to the overall results (Table 3).

**Table 3:** Total Thyroid Cancer and Papillary Thyroid Cancer Risk in NLST and PLCO by randomization group.

National Lung Screening Trial (NLST)						
Total Thyroid Cancer	Randomization Groups					
	**Overall**		**Male**		**Female**	
	**Control**	**Intervention**	**Control**	**Intervention**	**Control**	**Intervention**
Overall number of cases	23	37	11	17	12	20
Age-adjusted HR^1^ (95% CI)	1.00	1.61 (0.96, 2.71)	1.00	1.55 (0.72, 3.30)	1.00	1.66 (0.81, 3.40)
Active Screening HR^1^^,^ 2 (95% CI)	1.00	2.19 (1.07, 4.47)	1.00	2.55 (0.79, 7.98)	1.00	2.00 (0.81, 4.96)
Passive Follow-up HR^1^^,^ 2 (95% CI)	1.00	1.08 (0.49, 2.37)	1.00	1.00 (0.35, 2.84)	1.00	1.19 (0.36, 3.90)
**Papillary Thyroid**	**Overall**		**Male**		**Female**	
	**Control**	**Intervention**	**Control**	**Intervention**	**Control**	**Intervention**
Overall number of cases	16	28	7	9	9	11
Age-adjusted HR^1^ (95% CI)	1.00	1.25 (0.64, 2.41)	1.00	1.29 (0.47, 3.45)	1.00	1.22 (0.50, 2.94)
Active Screening HR^1^^,^ 2 (95% CI)	1.00	1.76 (0.74, 4.18)	1.00	1.67 (0.40, 6.99)	1.00	1.80 (0.60, 5.38)
Passive Follow-up HR^1^^,^ 2 (95% CI)	1.00	0.75(0.26, 2.16)	1.00	1.00(0.25, 3.99)	1.00	0.50(0.09, 2.71)

Prostate, Lung, Colorectal, and Ovarian Cancer Screening Trial (PLCO)						
Total Thyroid Cancer	Randomization Groups					
	**Overall**		**Male**		**Female**	
	**Control**	**Intervention**	**Control**	**Intervention**	**Control**	**Intervention**
Overall number of cases	130	104	53	34	77	70
Age-adjusted HR^1^ (95% CI)	1.00	0.79 (0.61, 1.02)	1.00	0.61 (0.40, 0.95)	1.00	0.91 (0.66, 1.26)
Active Screening HR^1^^,^ 2 (95% CI)	1.00	0.70 (0.46, 1.05)	1.00	0.44 (0.22, 0.90)	1.00	0.90 (0.54, 1.51)
Passive Follow-up HR^1^^,^ 2 (95% CI)	1.00	0.85 (0.61, 1.19)	1.00	0.76 (0.44, 1.31)	1.00	0.91 (0.62, 1.39)
**Papillary Thyroid**	**Overall**		**Male**		**Female**	
	**Control**	**Intervention**	**Control**	**Intervention**	**Control**	**Intervention**
Overall number of cases	106	75	41	22	65	53
Age-adjusted HR^1^ (95% CI)	1.00	0.70 (0.52, 0.94)	1.00	0.51 (0.31, 0.86)	1.00	0.82 (0.57, 1.17)
Active Screening HR^1^^,^ 2 (95% CI)	1.00	0.65 (0.41, 1.04)	1.00	0.45 (0.20, 1.05)	1.00	0.78 (0.44, 1.38)
Passive Follow-up HR^1^^,^ 2 (95% CI)	1.00	0.73 (0.50, 1.07)	1.00	0.56 (0.29, 1.07)	1.00	0.84 (0.53, 1.35)

1 Adjusted for age, sex (overall).

2 Uses a time dependent variable with cut point at 3 years (NLST) & 6 years (PLCO).

A significant increase in thyroid cancer risk was observed during active follow-up (HR  = 2.19; 95% CI: 1.07–4.47) in NLST but not in the following years (HR  = 1.08; 95% CI: 0.49–2.37); however, we did not find evidence of an interaction by period of follow-up (p = 0.19). The strength and direction of the association were similar during active follow-up for men (HR  = 2.55; 95% CI: 0.79–7.98) and women (HR  = 2.00; 95% CI: 0.81–4.96). Our results for papillary thyroid cancer during both time periods were similar to those observed for total thyroid, though slightly attenuated (Table 3).

Comparing the screened versus the unscreened arm in PLCO we observed a non-significant decrease in risk of total thyroid cancer (HR  = 0.70; 95% CI: 0.46–1.05) and papillary thyroid cancer (HR  = 0.65; 95% CI: 0.41–1.04) during active follow-up. The non-significant decrease in total thyroid cancer risk, while attenuated, persisted after the active follow-up period with no evidence of an interaction by period of follow-up (p =  .46). Results during active follow up were strongest, and statistically significant, for total thyroid cancer in men (HR = 0.44; 95% CI: 0.22–0.90). There was no clear association between screening and thyroid cancer risk in women during either follow-up period (Table 3).

## Discussion

The trends in thyroid cancer incidence over the past three decades are at least partially a result of increased diagnostic imaging and screening activities [18]–[21]. Previous studies into thyroid cancer trends show an increase in the use of medical imaging overall and in diagnosis of thyroid disorders [18], [22], [23], a disproportionate increase in the diagnosis of small micro-carcinomas of the thyroid [24]–[26], and a correlation between access to healthcare and thyroid cancer [9], [10]. These findings suggest that certain medical encounters may be implicated in thyroid cancer trends.

In our study of two large randomized cancer screening trials we found that the association with randomization to the intervention arm was different for each of the trials. For NLST, we observed that active screening via CT scan for lung cancer was associated with increased risk of thyroid cancer overall and the papillary subtype. This result was in agreement with our primary hypothesis that medical encounters which use highly sensitive medical imaging may be more likely to detect incidental thyroid cancers. In PLCO we observed that active screening for prostate, lung, colorectal and/or ovarian cancer was associated with lower risk of thyroid cancer overall and the papillary subtype. Because the medical encounters for prostate, colorectal, and ovarian cancer did not image or directly evaluate the thyroid gland, we hypothesized no association between screening for these cancers and risk of thyroid cancer. While the imaging field of the chest x-rays utilized for lung cancer screening may have included the thyroid gland, it was unclear how this would be associated with thyroid cancer because of the low resolution of the chest x-ray. Possible explanations for the results observed in each study are discussed below.

In NLST, screening for lung cancer was performed comparing two methods: low-dose helical CT scan in the intervention group and chest x-ray in the control group [11]. Randomization to receive screening via low dose helical CT scans may have been associated with thyroid cancer because this form of imaging is highly sensitive and images the portion of the neck where the thyroid gland is located. If an abnormality other than a suspected lung cancer (such as a suspected thyroid cancer) was discovered, this abnormality would have been noted and the study participant would have been referred to their primary care provider for follow-up [27]. The results of our analysis of NLST correspond to other studies which document a higher proportion of thyroid cancers detected by CT scans than other imaging modalities, including x-rays, because of their higher resolution [28]. Our results suggest that it may be important for clinicians to consider the costs and benefits of further work-ups for thyroid nodules detected incidentally from screening or other diagnostic procedures. Others have noted that over-diagnosis leads to over-treatment, where patients are subjected to the potential harms and increased medical costs related to treatments for conditions which may have caused them little-to-no harm [29].

Discussion of over-diagnosis and over-treatment can be a contentious issue at times. Evidence of this comes from the public outcry to changes in guidelines for breast and prostate cancer screening by the United States Preventive Services Task Force (USPSTF) which highlighted the need for an efficient and transparent recommendation process for screening [30]. Esserman et al. [31] attempt to outline a clear process by which improvements in areas where over-diagnosis and over-treatment is occurring can be made without sacrificing the effectiveness of cancer screening and treatment strategies. Recent efforts at highly individualized therapy for differentiated thyroid cancer [32] highlights the potential for researchers and clinicians to learn from the past over-diagnosis and over-treatment of breast and prostate cancer, as well as guidelines from researchers such as Esserman et al.

While the results for NLST agreed with our hypothesis, those for PLCO did not. There was an inverse association between randomization to the intervention arm and thyroid cancer risk which appeared to be driven by the results for men. Screening was associated with lower thyroid cancer risk in men during both the active and follow-up periods. Given our results in PLCO there had to be either a greater number of cancer cases detected in the control group than expected or a lower than expected number of cancer cases detected in the intervention group, or both. The 13 years of follow-up for individuals in this study occurred between 1997 and 2009. Total thyroid and papillary thyroid cancer rates for men ages 55–74 during this time period would have been approximately 8.5 to 15.2 per 100,000 person-years and 6.2 to 12.6 per 100,00 person-years respectively [33]. The actual rates of total thyroid (12.5 per 100,000 person-years) and papillary thyroid (9.7 per 100,000 person-years) cancer for men in the control arm are within this range, whereas the actual rates of thyroid (7.8 per 100,000 person-years) and papillary thyroid (5.1 per 100,000 person-years) cancer for men in the intervention arm were lower than expected. These data suggest that there is little evidence for a surplus of thyroid cancer in the control group but some evidence for a lower than expected number of thyroid cancer cases in the intervention group.

Men and women in the intervention arm of PLCO were screened for three types of cancer, while controls did not receive any additional care. One possible explanation for the observed results is that participants in the intervention arm, may have felt they were receiving all of the medical care they needed and then may have been less likely to seek outside medical care during the course of the trial. Additionally, the physicians in the intervention group were providing screening for PLCO cancers and were not instructed to perform other primary care assessments. This could have resulted in fewer thyroid cancers detected incidentally than expected for men by either PLCO physicians or the primary care physicians of men in the intervention arm. Evidence to support this hypothesis would have to come from information on stage and size of thyroid tumors at diagnosis indicate that the deficit in the number of thyroid cancers we observed were for small and early-stage tumors. Unfortunately, information on stage and size is not available for this trial. Additionally, this explanation does not explain why we did not observe the same pattern in women as we did for men. As thyroid cancer trends, particularly in women, have gained national attention more women may be having their thyroids checked at an annual gynecological appointment, for which there is no male equivalent.

Because of the incidence for prostate, lung, and ovarian cancer, were greater in the intervention arm than the control arm, a second possible explanation for the observed results is that informative censoring occurred in the intervention arm of PLCO. A primary assumption of survival analysis is one of non-informative censoring. If the assumption of non-informative censoring is violated then the estimators of covariate effects are biased [34]. The implications of informative censoring would be that individuals in the intervention arm were diagnosed at higher rates for prostate, lung, and ovarian cancers and were then not available for diagnosis of a first primary thyroid cancer. This would lead to fewer thyroid cancer cases than expected in the intervention arm. However, the real effects of informative censoring on our results are likely to be minimal. There were approximately 400 additional prostate cancers diagnosed and 1,000 additional lung cancers diagnosed in the intervention arm of PLCO than in the control arm. Because thyroid cancer was rare for men in this trial (12.5 per 100,000 person-years) this means informative censoring would account for approximately two fewer thyroid cancer cases in the intervention arm of the trial (12.5 per 100,000 person-years ×1,400 people ×11 years median follow-up time  = 1.93). As this is much lower than the twenty-six fewer cases of thyroid cancer observed in the intervention arm of PLCO, informative censoring is not a likely explanation for our findings.

There were several advantages of our study including randomization of the study exposure, use of only confirmed thyroid cancers, available information on thyroid cancer histology, and use of a time dependent covariate which allowed us to see how the HR for thyroid cancer differed before and after the active screening period. Additionally, our study is the first of its kind to assess the effect of screening on thyroid cancer prospectively using large randomized screening trials. Our analysis was not without limitations, the most important of which was the relatively small number of thyroid cancer cases in each trial, particularly NLST. This made it difficult to estimate the risk of thyroid cancer with high precision, especially when attempting to calculate the risk for the papillary thyroid cancer subtype. Another limitation is that we did not have information on thyroid tumor size, which would have allowed us to test whether the thyroid cancers in the intervention arm of NLST were smaller and of an earlier stage than in the control arm. Finally, it is possible that the results of our study may have been due to chance.

## Conclusions

The implications of our study are important. The complexity of cancer diagnosis as well as the need to better understand over-diagnosis and over-treatment have gained national attention [35]. Our study suggests that the types of medical encounters, and not just the number of encounters, are important factors in whether an individual will receive a thyroid cancer diagnosis. Specifically, encounters that may lead to over-diagnosis seem to occur in scenarios where highly sensitive medical imaging, such as CT scans, are used to image areas including the neck. There is no evidence from our study that screening activities which do not result in the imaging of the thyroid should be of concern when discussing thyroid cancer over-diagnosis. However, palpation of the neck is commonly used by physicians to detect thyroid cancers and was not done in either arm of NLST or PLCO, so the impact of palpation could not be assessed in our trial.

One area where our results could be of clinical importance is in how to proceed when a patient is diagnosed with thyroid cancer. Currently there is some controversy regarding the best course of action for patients diagnosed with thyroid cancer because a small proportion of papillary micro-carcinomas are aggressive and it is difficult to predict which will be aggressive. Primary treatment of thyroid cancer involves removing the thyroid surgically, which exposes patients to the risks associated with surgical procedures and also necessitates a lifetime of thyroid hormone supplementation [36], [37]. Many clinicians support the use of total thyroidectomy because of high rates of recurrence seen in patients treated with less radical thyroid lobectomy [38], [39], while others have seen success with watchful waiting [40].

If imaging reveals a potential thyroid lesion it is important for the patient, and clinician together, to make a decision on how to proceed using all the available information. Understanding the role of imaging in over-diagnosis and the potential to over-treat in this scenario may cause them to take one of the more conservative courses of action being advocated, such as watchful waiting [40], [41]. Future studies, using genetic markers, would be helpful in distinguishing truly incidental thyroid carcinomas from more aggressive ones. Two areas of potential interest are the findings that *BRAF* mutations may be useful in predicting papillary thyroid cancer aggressiveness [42], [43] and the use of microRNA expression patterns to predict thyroid cancer recurrence after surgery [44].

Our study could be used in the future, with other recent studies which have investigated whether all papillary thyroid micro-carcinomas should be aggressively treated [45], to create a clinical checklist indicating which patients would be good candidates for watchful waiting. Future randomized trials could compare immediate treatment via total thyroidectomy with watchful waiting on the risk of thyroid cancer metastasis or other outcomes. Finally, as some have suggested, one possibility would be to re-classify incidentally detected micro-carcinomas of the thyroid as micro-papillary lesions of indolent course [29]. Re-classification of thyroid micro-carcinomas, to a non-cancerous disease, may help convey a favorable prognosis to the patient, avoid over-treatment, and improve a patient's receptiveness to watchful waiting.
